# Naloxone reversal of the cardiorespiratory depressant effects of opioids and mixtures of opioids and stimulants in rats

**DOI:** 10.3389/fphar.2025.1654791

**Published:** 2025-09-22

**Authors:** Jacqueline M. Vazquez, Robert W. Seaman, Drew Caldron, Gregory T. Collins

**Affiliations:** ^1^ Department of Pharmacology, University of Texas Health San Antonio, San Antonio, TX, United States; ^2^ South Texas Veterans Healthcare System, San Antonio, TX, United States

**Keywords:** polysubstance abuse, opioids, stimulants, naloxone, pulse oximetry

## Abstract

Co-use of opioids (e.g., fentanyl) and stimulants (e.g., methamphetamine; METH) contributed to >30% of the almost 106,000 fatal overdoses in the United States in 2023. Although NarCan^®^ (naloxone) is effective at reversing opioid-induced cardiorespiratory depression, larger and/or more frequent doses are often required for fentanyl and multi-drug overdoses involving fentanyl. Using collar-based pulse oximetry, this study characterized the effects of intravenous (IV) fentanyl (0.0056–0.56 mg/kg), heroin (0.32–5.6 mg/kg), and METH (0.1–1 mg/kg), as well as mixtures of 0.56 mg/kg fentanyl +1 mg/kg METH and 5.6 mg/kg heroin +1 mg/kg METH on blood oxygen saturation (SpO_2_), heart rate (HR), and breath rate (BR) in male and female Sprague-Dawley rats. To evaluate the potency and effectiveness of naloxone to reverse cardiorespiratory depression, naloxone (0.01–3.2 mg/kg; IV) or vehicle was administered 5 min after opioids or opioid + stimulant mixtures. Naloxone was fully effective at reversing the effects of fentanyl and heroin alone but was more potent for fentanyl. Naloxone was fully effective and equipotent at reversing the cardiorespiratory effects of heroin and heroin + METH but was less potent and less effective at reversing the cardiorespiratory effects of fentanyl + METH compared to fentanyl alone. When administered after fentanyl, heroin, or heroin + METH, naloxone recovered baseline SpO_2_ in all rats, however, SpO_2_ was only recovered in 75% of rats treated with fentanyl + METH. These findings suggest that naloxone may be less potent and effective at reversing fentanyl-induced cardiorespiratory depression when METH is co-administered.

## 1 Introduction

The United States has been battling an opioid epidemic involving multiple waves (e.g., prescription opioids, heroin, fentanyl) for the past 30+ years (Han et al., 2019; Ciccarone, 2021). Of the almost 106,000 fatal overdoses in the United States in 2023, nearly 70% were attributed to opioids, primarily fentanyl and fentanyl analogues (Hedegaard et al., 2020). However, it is becoming increasingly recognized that the overdose epidemic is now driven by multi-drug overdoses (e.g., fentanyl with stimulant drugs) (Ellis et al., 2018; Jones et al., 2020; Ciccarone, 2021; Friedman and Shover, 2023). Of the nearly 73,000 synthetic opioid overdose deaths in the United States in 2023, approximately 50% also involved a stimulant (e.g., methamphetamine [METH], cocaine) (National Institute on Drug Abuse, 2024). Cardiorespiratory depression is the primary cause of death for opioid overdoses (White and Irvine, 1999; Dahan et al., 2010), and although there are effective treatments for reversing opioid-induced cardiorespiratory depression (e.g., naloxone; NarCan^®^), clinical evidence suggests that larger and/or more frequent doses are required for fentanyl and multi-drug overdoses involving fentanyl (Mayer et al., 2018; Pergolizzi Jr et al., 2021a; van Lemmen et al., 2023; Dahan et al., 2024). However, administering larger doses of naloxone also increases risk for adverse cardiorespiratory effects (e.g., tachycardia, tachypnea), which may interact with the cardiovascular effects of stimulants (Kanof et al., 1992; Merigian, 1993; Hunter, 2005; Lameijer et al., 2014; Yugar et al., 2023). Thus, multi-drug overdoses pose a significant public health problem that requires further investigation into the factors (e.g., opioid vs. opioid + stimulant) that might impact the potency and effectiveness of naloxone to safely reverse overdoses involving opioids and stimulants.

Opioid-induced cardiorespiratory depression is mediated by activation of mu opioid receptors (MORs) in brainstem respiratory centers and is characterized by decreased heart rate and ventilation, ultimately resulting in decreased blood oxygenation (i.e., hypoxemia) (Lalley, 2003; Montandon et al., 2011; Zhang et al., 2011; Liu et al., 2021; Bateman et al., 2023; Kiyatkin and Choi, 2024). Naloxone reversal of cardiorespiratory depression can precipitate withdrawal at large doses in opioid-dependent individuals or those acutely intoxicated with opioids, which can present as psychological (e.g., irritability, aggression) and physiological (e.g., tachycardia, hypertension, tachypnea) signs and symptoms (Heishman et al., 1989; Kanof et al., 1992; Schulteis et al., 1994; Weisshaar et al., 2020; Purssell et al., 2021; Lewter et al., 2022). Many of these adverse effects, including rebound cardiorespiratory responses, are caused by increased norepinephrine levels in the locus coeruleus (Chang and Dixon, 1990; Feria et al., 1990; Brent and Chahl, 1991; Nestler et al., 1994; Maldonado, 1997; Kwok et al., 2024). Similarly, amphetamine-type stimulants produce tachycardia and hypertension through indirect activation of noradrenergic receptors (Schindler et al., 1992; Ferrucci et al., 2013; Ferrucci et al., 2019; Hassan et al., 2015; Neumann et al., 2023). As such, stimulant involvement in opioid overdoses may exacerbate increases in cardiovascular function commonly observed following reversal of opioid overdoses by naloxone. Case reports also suggest that naloxone reversal of multi-drug overdoses involving opioids and stimulants can result in severe cardiovascular complications (e.g., pulmonary edema, ventricular tachycardia, etc.) (Merigian, 1993; Hunter, 2005). Thus, it is important to understand how co-use of opioids and stimulants impacts the potency and effectiveness of naloxone to reverse cardiorespiratory depression.

In addition to challenges posed from the co-involvement of stimulants in opioid overdoses, fentanyl also has unique properties that may further complicate reversal. Fentanyl produces a condition known clinically as “wooden chest syndrome”, which is characterized by rigidity of the intercostal muscles and diaphragm as well as vocal cord closure (Buxton et al., 2018; Torralva and Janowsky, 2019; Pergolizzi Jr et al., 2021c; Burgraff et al., 2023; Cavallo et al., 2023). Preclinical studies have shown that fentanyl-induced chest wall rigidity likely results from MOR-mediated increase in noradrenergic output from the locus coeruleus (Jerussi et al., 1987; Lui et al., 1989; Lui et al., 1993; Weinger et al., 1989; Weinger et al., 1995; Weinger and Taurek, 1990; Negus et al., 1993; Fu et al., 1994; Weinger and Bednarczyk, 1994; Campbell et al., 1995; Lee et al., 1995; Haouzi and Tubbs, 2022). Doses of fentanyl larger than 0.025 mg/kg have also been shown to produce persistent and naloxone-resistant vocal cord closure in rats, whereas a smaller fentanyl dose (0.005 mg/kg) and large morphine dose (5 mg/kg in rats) only produced brief laryngospasm that fully resolved (Miner et al., 2021). Because these potentially naloxone-insensitive effects of fentanyl involve noradrenergic systems they may increase the risk for adverse outcomes (e.g., pulmonary edema, ventricular tachycardia) when stimulants are co-used (Hunter, 2005; Purssell et al., 2021). Taken together, co-use of fentanyl and METH may decrease the potency and effectiveness of naloxone to safely reverse cardiorespiratory depression and result in sympathomimetic toxicity.

The current study sought to characterize the potency and effectiveness of naloxone to reverse cardiorespiratory depression induced by opioids alone (i.e., fentanyl, heroin) and opioid + stimulant mixtures (i.e., fentanyl + METH, heroin + METH). Collar-based pulse oximetry was used to test the hypotheses that: 1) naloxone will be less potent and/or effective at reversing cardiorespiratory depression from fentanyl than heroin, and 2) naloxone will be equipotent and/or effective at reversing cardiorespiratory depression from heroin alone and a mixture of heroin + METH, but less potent and/or effective at reversing cardiorespiratory depression from a mixture of fentanyl + METH than fentanyl alone.

## 2 Methods

### 2.1 Subjects

96 Sprague-Dawley rats (n = 48/sex, 225–250 g upon arrival for females, 275–300 g upon arrival for males) were purchased from Envigo (Indianapolis, IN, United States). Rats were individually housed in a temperature- and humidity-controlled room and maintained on a 14/10-h light/dark cycle. All experiments were conducted during the light cycle at approximately the same time each day. Rats were provided *ad libitum* access to Purina rat chow and water. All studies were carried out in accordance with the Institutional Animal Care and Use Committees of the University of Texas Health Science Center at San Antonio and the eighth edition of the Guide for Care and Use of Laboratory Animals (National Research Council, 2011).

### 2.2 Surgery

Rats were anesthetized with 2%–3% isoflurane and prepared with chronic indwelling catheters in the left femoral vein, as previously described (Doyle et al., 2021; Seaman and Collins, 2021). Briefly, a trocar was used to tunnel catheters under the skin that attached to a vascular access port placed in the mid-scapular region. Immediately following surgery, rats were administered Excede^®^ (20 mg/kg; SC) or Baytril (10 mg/kg; SC) to prevent infection and meloxicam (1 mg/kg; SC) to minimize pain and discomfort. Rats were allowed 5–7 days to recover and catheters were flushed daily with 0.5 mL of heparinized saline (100 U/mL). During experimentation, catheters were flushed daily with 0.2 mL of saline before and 0.5 mL of heparinized saline after pulse oximetry sessions.

### 2.3 Drugs

Fentanyl HCl and heroin HCl were generously provided by the National Institute on Drug Abuse Drug Supply Program (Bethesda, MD). D-methamphetamine HCl, naloxone HCl and naltrexone HCl were purchased from Sigma-Aldrich (St. Louis, MO, United States). All drugs were dissolved in physiological saline and passed through a 0.2 μm syringe filter prior to administration. For pulse oximetry sessions, all drugs were administered by intravenous (IV) infusion in a volume of 1 mL/kg and flushed with an infusion of 0.5 mL of saline.

### 2.4 Apparatus

All experiments were conducted in clear small animal enclosures located within ventilated, light- and sound-attenuated chambers (Med Associates, Inc., St. Albans, VT; STARR Life Sciences Corp., Oakmont PA). Each pulse oximetry enclosure was equipped with a swivel mount and lever arm with a low torque slip ring allowing for easy management of tethers and wires. Drug infusions were manually delivered through an infusion tether, which was attached to the pulse oximetry lead. Blood oxygen saturation (SpO_2_; %), heart rate (HR; beats/min), and breath rate (BR; breaths/min) were continuously recorded using the MouseOx Plus 2.0 Premium Software (STARR Life Sciences Corp., Oakmont PA).

### 2.5 Pulse oximetry

Prior to each session, rats were briefly anesthetized with 2%–3% isoflurane and an electric shaver was used to remove hair from around the neck, and to secure a pulse oximeter collar sensor around the rat’s neck. Each pulse oximetry session began with a 1-h habituation period, followed by two infusions, 5 minutes apart. After the second infusion, recordings continued for 25 min. Infusion one was saline (0.5 mL), fentanyl (0.0056–0.56 mg/kg), heroin (0.32–5.6 mg/kg), METH (0.1–1 mg/kg), or a mixture of either fentanyl (0.56 mg/kg) + METH (1 mg/kg), or heroin (5.6 mg/kg) + METH (1 mg/kg). Infusion two was either saline or naloxone (0.01–3.2 mg/kg). Each infusion was followed by 0.5 mL saline to ensure all drug was administered. Each data file was timestamped to ensure that the data corresponding to drug infusions could be located. After the session, animals were administered naltrexone (0.32 mg/kg) if SpO_2_ levels had not returned to ≥90%. The time frame between infusions (i.e., 5 min) was chosen to ensure the maximal effect of fentanyl or heroin on SpO_2_ would be reached and maintained for at least 1 minute prior to naloxone administration. To reduce the likelihood of tolerance developing to the effects of the opioids, animals were only tested once a week.

### 2.6 Quantification of data

For agonists alone, the primary dependent variables of cardiorespiratory depression were SpO_2_, HR, and BR. Opioid doses for antagonist reversal tests were selected based on the capacity to reduce SpO_2_ to ≥30% of baseline (i.e., saline–saline tests) for at least 30 min. Recovery of baseline SpO_2_ was defined as averaging 90% SpO_2_ for at least 5 minutes after the second infusion, with the first minute in the average recorded as the time to recover baseline SpO_2_. Primary dependent variables for antagonist reversal of cardiorespiratory depression were also SpO_2_, HR, and BR. Rebound tachycardia and rebound tachypnea were defined as the maximum HR or BR value recorded within 10 min after naloxone infusion minus the average HR or BR values over this same time frame (5–15 min) when the same subject was tested saline for both infusions.

### 2.7 Data analysis

Time-effect graphs produced from pulse oximetry readings were analyzed using area under the curve (AUC) analysis. Data were calculated for individual subjects and presented as the group mean ± the standard error of the mean (S.E.M.). For agonists alone (i.e., fentanyl-saline, heroin-saline), two-way ANOVAs (factors being opioid dose and sex) were performed to determine any statistically significant differences from baseline (i.e., saline-saline) in cardiorespiratory variables (e.g., SpO_2_, HR, BR). For antagonist reversal studies, two-way ANOVAs (factors being naloxone dose and sex) were performed to determine any statistically significant differences from baseline in cardiorespiratory variables. For analysis of recovery of baseline SpO_2_, a multiple logistic regression analysis using a variable slope (four parameters) model was performed to compare the respective opioid alone and opioid + stimulant mixture, with the logEC_50_ and Hill slope unconstrained and the other two parameters constrained as follows: (1) bottom, constant equal to 0, and (2) top, must be between zero and 100.1. Three-way ANOVAs (factors being opioid condition, naloxone dose, and sex) were also conducted to determine statistically significant differences in naloxone reversal of HR and BR (i.e., rebound tachycardia/tachypnea), and minutes to recovery between the respective opioid alone and opioid + stimulant mixture condition.

## 3 Results

### 3.1 Fentanyl and heroin dose-dependently produced cardiorespiratory depression

**FIGURE 1:**
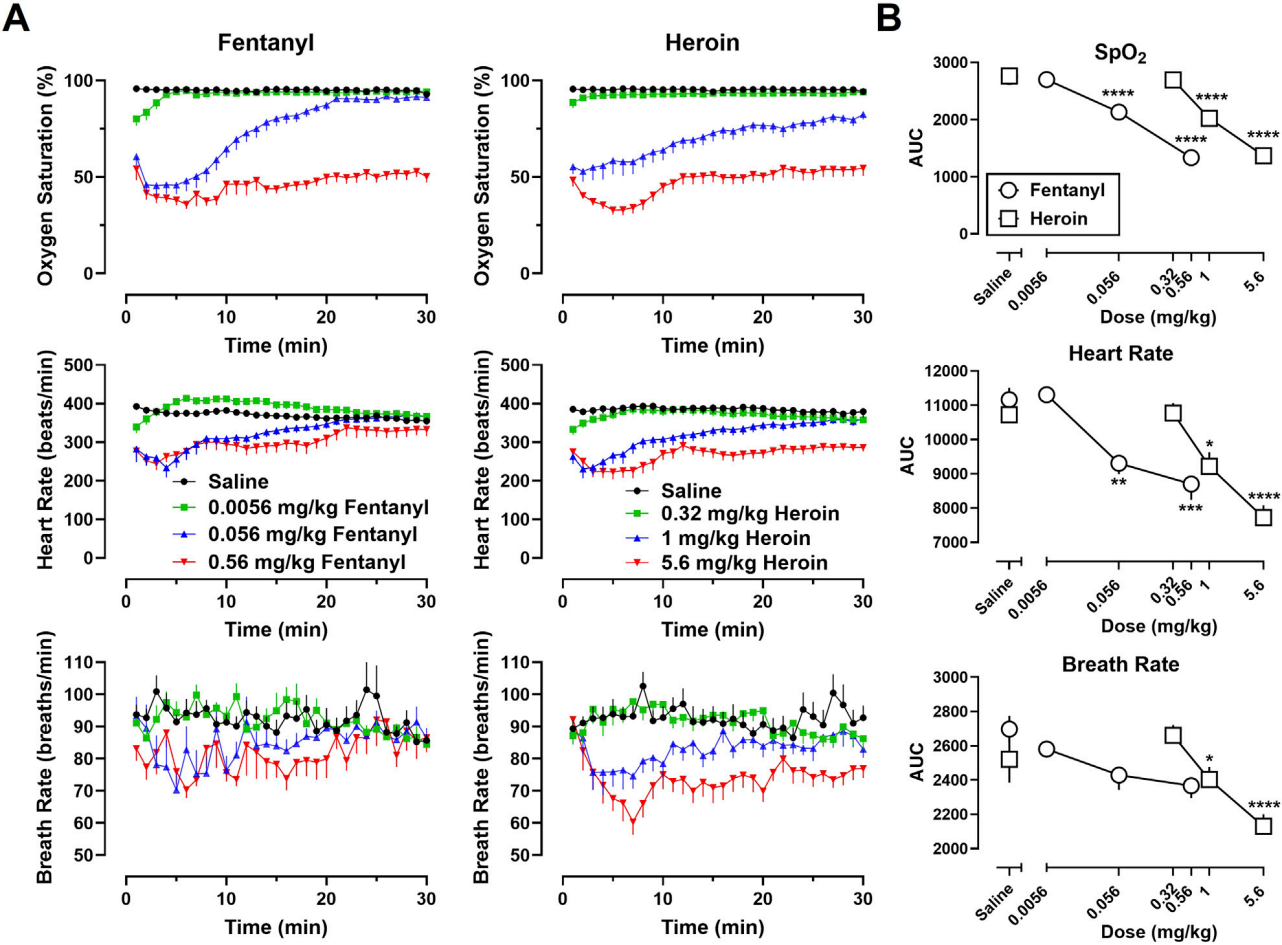
**(A)** Time-effect functions for fentanyl (0.0056–0.56 mg/kg) (left) and heroin (0.32–5.6 mg/kg) (right) on blood oxygen saturation (SpO_2_), heart rate, and breath rate. **(B)** Dose-effect functions for fentanyl and heroin are shown in the area under the curves. Data represent the mean (±SEM), and each symbol represents 12 rats (n = 6/sex). **** indicates p < 0.0001, *** indicates p < 0.001, ** indicates p < 0.01, and * indicates p < 0.05.

Time-effect functions were established for fentanyl alone (Figure 1A, left) and heroin alone (Figure 1A, right) on SpO_2_, HR, and BR. The AUC analyses for the time-effect functions (Figure 1B) demonstrate that heroin produced dose-related decreases in SpO_2_ (F [3, 30] = 99.97; p < 0.0001), HR (F [3, 30] = 27.84; p < 0.0001), and BR (F [3, 30] = 17.04; p < 0.0001); there were no significant effects of sex, or dose × sex interactions. Post-hoc analyses indicate that 1 mg/kg heroin and 5.6 mg/kg heroin differed significantly from saline for SpO_2_ (p < 0.0001 for both), HR (p < 0.05 and p < 0.0001, respectively), and BR (p < 0.05 and p < 0.0001, respectively). Fentanyl produced dose-related decreases in SpO_2_ (F [3, 30] = 161.5; p < 0.0001) and HR (F [3, 30] = 18.45; p < 0.0001), but not BR (F [3, 30] = 1.25; p = 0.31); there were no significant effects of sex, or dose × sex interactions. Post-hoc analyses indicate that 0.056 mg/kg fentanyl and 0.56 mg/kg fentanyl differed significantly from saline for SpO_2_ (p < 0.0001 for both) and HR (p < 0.01 and p < 0.0001, respectively).

### 3.2 Naloxone reversal of the cardiorespiratory depressant effects of fentanyl and heroin alone

**FIGURE 2:**
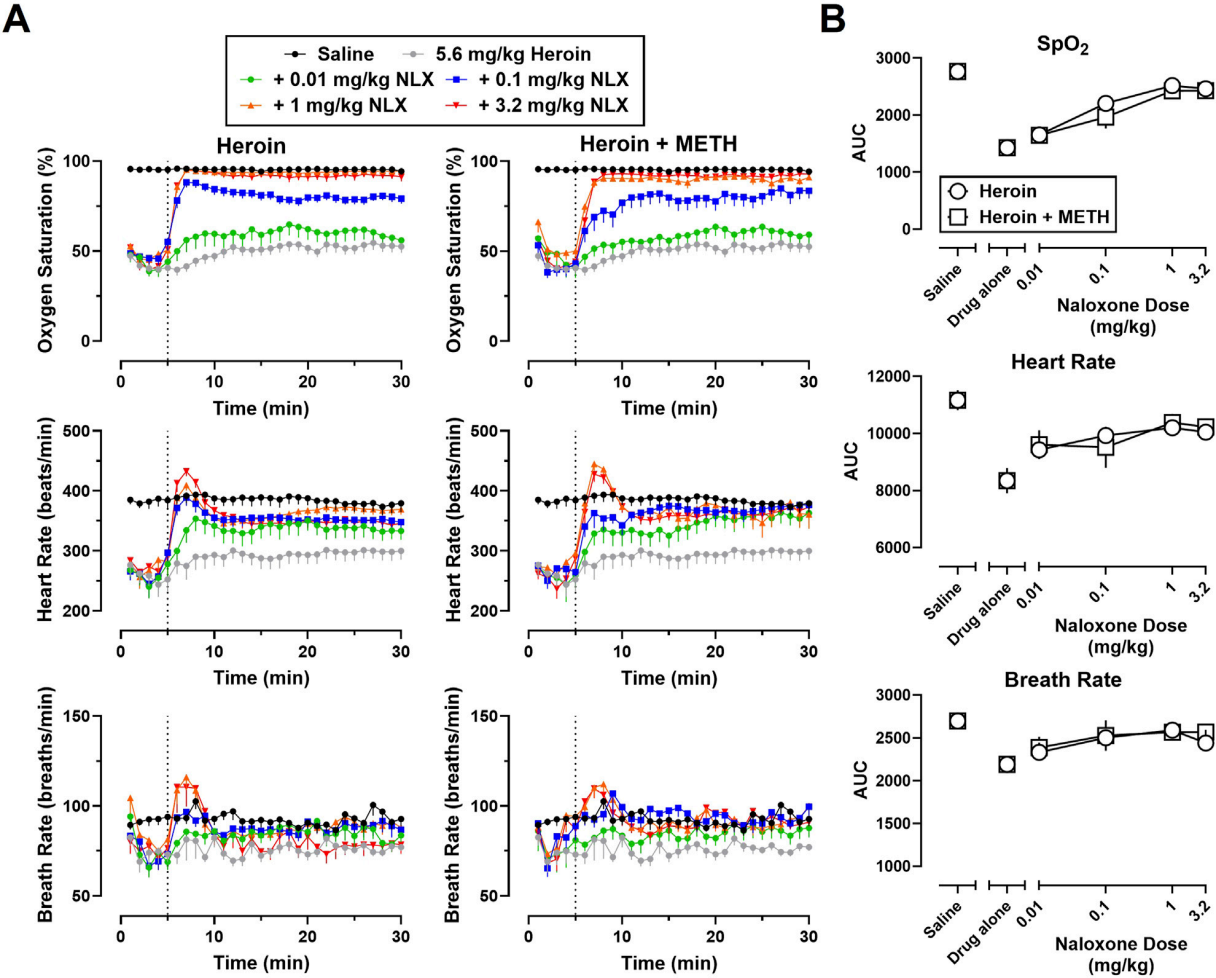
**(A)** Time-effect functions for naloxone (0.01–3.2 mg/kg) reversal of heroin (5.6 mg/kg) (left) and 5.6 mg/kg heroin +1 mg/kg METH (right) on blood oxygen saturation, heart rate, and breath rate. **(B)** Dose-effect functions for naloxone reversal of heroin and heroin + METH are shown in the area under the curves. Data represent the mean (±SEM), and each symbol represents 12 rats (n = 6/sex). The vertical grey dotted line represents the 5-min timepoint after the first infusion where naloxone (0.01–3.2 mg/kg) or vehicle was administered.

**FIGURE 3:**
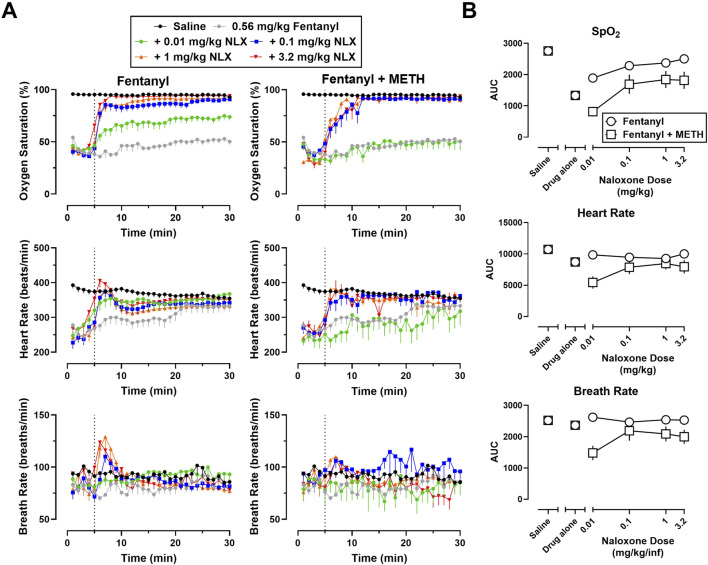
**(A)** Time-effect functions for naloxone (0.01–3.2 mg/kg) reversal of fentanyl (0.56 mg/kg) (left) and 0.56 mg/kg fentanyl +1 mg/kg METH (right) on blood oxygen saturation, heart rate, and breath rate. **(B)** Dose-effect functions for naloxone reversal of fentanyl and fentanyl + METH are shown in the area under the curves. Data represent the mean (±SEM), and each symbol represents 12 rats (n = 6/sex). The vertical grey dotted line represents the 5-min timepoint after the first infusion where naloxone (0.01–3.2 mg/kg) or vehicle was administered.

Time-effect functions were established for naloxone (0.01–3.2 mg/kg) reversal of the cardiorespiratory depressant effects of heroin (5.6 mg/kg) alone (Figure 2A, left) and fentanyl (0.56 mg/kg) alone (Figure 3A, left) on SpO_2_, HR, and BR. The AUC analyses for the time-effect functions (Figure 2B) demonstrate that naloxone dose-dependently reversed the depressant effects of heroin alone on SpO_2_ (F [5, 60] = 45.77; p < 0.0001), HR (F [5, 60] = 7.49; p < 0.0001), and BR (F [5, 60] = 3.24; p < 0.05); there were no significant effects of sex, or dose × sex interactions. The AUC analyses for the time-effect functions (Figure 3B) demonstrate that naloxone dose-dependently reversed the depressant effects of fentanyl on SpO_2_ (F [5, 60] = 56.62; p < 0.0001) and HR (F [5, 60] = 4.31; p < 0.01), but not BR (F [5, 60] = 0.76; p = 0.58); there were no significant effects of sex, or dose × sex interactions.

**FIGURE 4:**
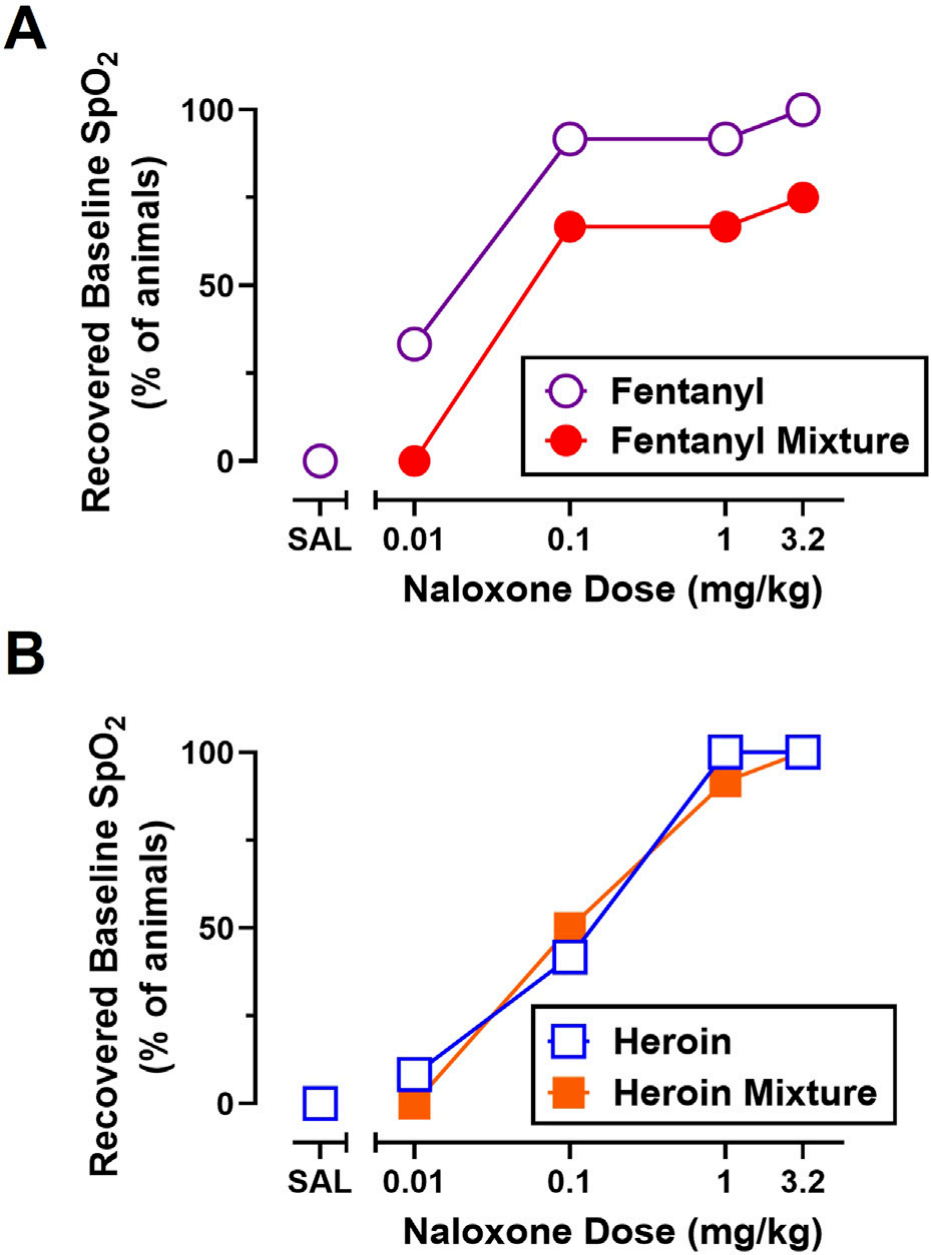
Effects of naloxone (0.01–3.2 mg/kg) administration on the percent of animals that recovered baseline blood oxygen saturation (SpO_2_) from fentanyl (0.56 mg/kg) and 0.56 mg/kg fentanyl +1 mg/kg METH **(A)** and heroin (5.6 mg/kg) and 5.6 mg/kg heroin +1 mg/kg METH **(B)**. Data represent percentage values, and each symbol represents 12 rats (n = 6/sex).

Recovery of baseline SpO_2_ from fentanyl (0.56 mg/kg) and heroin (5.6 mg/kg) in the absence of naloxone (i.e., fentanyl-saline, heroin-saline) can be seen in Figures 4A,B, respectively. As shown in Figure 4, naloxone (0.01–3.2 mg/kg) produced dose-related increases in the percentage of animals that met recovery criteria for fentanyl and heroin alone. A multiple logistics regression analysis revealed that one curve could not fit the recovery data for fentanyl and heroin alone (p < 0.05) and that naloxone was more potent at reversing cardiorespiratory depression from fentanyl alone (ED_50_ = 0.016) than heroin alone (ED_50_ = 0.13).

The time in minutes for animals to recover ≥90% SpO_2_ when fentanyl (0.56 mg/kg) and heroin (5.6 mg/kg) were followed by saline or naloxone (0.01–3.2 mg/kg) are shown in Table 1. Without naloxone, 0% of animals recovered baseline SpO_2_ following treatment with 0.56 mg/kg fentanyl or 5.6 mg/kg heroin. A three-way ANOVA for fentanyl alone and heroin alone revealed a main effect of naloxone dose (F [3, 80] = 34.22; p < 0.0001) and sex (F [1, 80] = 4.73; p < 0.05) on time to recovery, with female rats recovering faster and at smaller doses than males. A dose of 3.2 mg/kg naloxone recovered SpO_2_ within 1–2 min; there were no significant effects of opioid (i.e., fentanyl or heroin) or any interactions.

**TABLE 1:** Effects of naloxone (0.01–3.2 mg/kg) administration on the time in minutes it took for animals to recover baseline blood oxygen saturation (SpO_2_) from heroin (5.6 mg/kg), 5.6 mg/kg heroin +1 mg/kg METH, fentanyl (0.56 mg/kg), and 0.56 mg/kg fentanyl +1 mg/kg METH. Data represent the mean (±SEM) and are presented as group average (n = 12) or by sex (n = 6/sex).

Drug condition and naloxone dose (mg/kg)	Time (mins) to recovery of baseline SpO_2_ (±S.E.M.) – Proportion of animals that recovered (n of 12)		
	Grouped	Males	Females
Heroin			
0	N/A – 0 of 12	N/A – 0 of 6	N/A – 0 of 6
0.01	2.00 (0) – 1 of 12	N/A – 0 of 6	2.00 (0) – 1 of 6
0.1	1.20 (0.20) – 5 of 12	1.00 (0) – 1 of 6	1.25 (0.25) – 4 of 6
1.0	1.25 (0.13) – 12 of 12	1.17 (0.17) – 6 of 6	1.33 (0.21) – 6 of 6
3.2	1.83 (0.42) – 12 of 12	2.33 (0.80) – 6 of 6	1.33 (0.21) – 6 of 6
Heroin + METH			
0	N/A	N/A	N/A
0.01	N/A – 0 of 12	N/A – 0 of 6	N/A – 0 of 6
0.1	2.67 (0.80) – 6 of 12	1.50 (0.50) – 2 of 6	3.25 (1.11) – 4 of 6
1.0	3.81 (1.58) −11 of 12	6.20 (3.32) – 5 of 6	1.3 (0.31) – 6 of 6
3.2	2.58 (1.05) – 12 of 12	1.50 (0.22) – 6 of 6	3.67 (2.08) – 6 of 6
Fentanyl			
0	N/A – 0 of 12	N/A – 0 of 6	N/A – 0 of 6
0.01	4.75 (3.09) – 4 of 12	2.00 (0) – 1 of 6	5.67 (4.18) – 3 of 6
0.1	6.81 (2.45) – 11 of 12	10.00 (3.93) – 6 of 6	3.00 (1.76) – 5 of 6
1.0	3.46 (1.32) – 11 of 12	1.80 (0.37) – 5 of 6	4.83 (2.33) – 6 of 6
3.2	1.17 (0.11) – 12 of 12	1.00 (0) – 6 of 6	1.33 (0.21) – 6 of 6
Fentanyl + METH			
0	N/A	N/A	N/A
0.01	N/A – 0 of 12	N/A – 0 of 6	N/A – 0 of
0.1	3.00 (1.31) – 8 of 12	5.00 (3.51) – 3 of 6	1.80 (0.37) – 5 of 6
1.0	1.38 (0.26) – 8 of 12	1.00 (0) – 4 of 6	1.75 (0.48) – 4 of 6
3.2	1.56 (0.29) – 9 of 12	1.75 (0.48) – 4 of 6	1.40 (0.40) – 5 of 6

### 3.3 Naloxone reversal of the cardiorespiratory depressant effects of fentanyl or heroin in a mixture with METH

Time-effect functions were established for METH alone (0.01–1 mg/kg) on SpO_2_, HR, and BR; however, the locomotor stimulant effects of METH resulted in poor signal quality from the pulse oximeter, and thus, a great deal of missing data and large variability for all three endpoints (data not shown). The largest dose of METH (1 mg/kg) was chosen for use in mixtures as it has been previously shown to increase HR and blood pressure (Arora et al., 2001; Hassan et al., 2016). Time-effect functions were then established for naloxone (0.01–3.2 mg/kg) reversal of the cardiorespiratory depressant effects of a mixture of 5.6 mg/kg heroin +1 mg/kg METH (Figure 2A, right) and a mixture of 0.56 mg/kg fentanyl +1 mg/kg METH (Figure 3A, right) on SpO_2_, HR, and BR. Naloxone reversal of heroin + METH produced dose-related increases in SpO_2_ (F [5, 60] = 23.82; p < 0.0001), HR (F [5, 60] = 5.13; p < 0.001), and BR (F [5, 60] = 2.72; p < 0.05); there was a small but significant main effect of sex for naloxone reversal of HR (F [1, 60] = 4.08; p < 0.05), with a greater reversal effect observed in males compared to females. Naloxone reversal of fentanyl + METH produced dose-related increases in SpO_2_ (F [5, 60] = 8.88; p < 0.0001), HR (F [5, 60] = 3.72; p < 0.01), and BR (F [5, 60] = 2.68; p < 0.05); there were no significant effects of sex, or dose × sex interactions.

As shown in Figure 4, naloxone (0.01–3.2 mg/kg) produced dose-related increases in the percentage of animals that met recovery criteria for all opioid alone and opioid + stimulant mixture conditions. Though 100% of rats recovered to ≥90% SpO_2_ when naloxone was administered after fentanyl alone, heroin alone, and heroin + METH, only 75% of rats recovered baseline SpO_2_ when naloxone was administered after a mixture of fentanyl + METH. A multiple logistics regression analysis revealed that one curve fits the recovery data for heroin alone and heroin + METH (p = 0.71) and that naloxone was equipotent at reversing cardiorespiratory depression from heroin alone (ED_50_ = 0.13) and heroin + METH (ED_50_ = 0.10). A multiple logistics regression analysis revealed that one curve could not fit for the recovery data for fentanyl alone and fentanyl + METH (p < 0.05) and that naloxone was ∼3.6-fold less potent at reversing cardiorespiratory depression from fentanyl + METH (ED_50_ = 0.044) than fentanyl alone (ED_50_ = 0.012).

The time in minutes for animals to recover to ≥90% SpO_2_ for at least 5 minutes after mixtures of fentanyl + METH and heroin + METH were followed by saline or naloxone (0.01–3.2 mg/kg) are shown in Table 1. A three-way ANOVA for heroin alone and heroin + METH revealed a main effect of naloxone dose (F [3, 80] = 54.40; p < 0.0001) and sex (F [1, 80] = 6.54; p < 0.05) on time to recovery, with female rats recovering faster and at smaller doses than males. A dose of 3.2 mg/kg naloxone recovered SpO_2_ within 2–3 min; there were no significant effects of drug condition (i.e., heroin vs. heroin + METH). A three-way ANOVA for fentanyl alone and fentanyl + METH revealed a main effect of naloxone dose (F [3, 80] = 17.26; p < 0.0001) and drug condition (F [1, 80] = 6.71; p < 0.05) on time to recovery; there was no significant effects of sex or any interactions. At a dose of 3.2 mg/kg naloxone, it took ∼7-fold longer for animals to recover from a mixture of fentanyl + METH (∼7 min) than fentanyl alone (∼1 min).

### 3.4 Naloxone dose-dependently increased rebound tachycardia and tachypnea from fentanyl and heroin alone and in a mixture with METH

**FIGURE 5:**
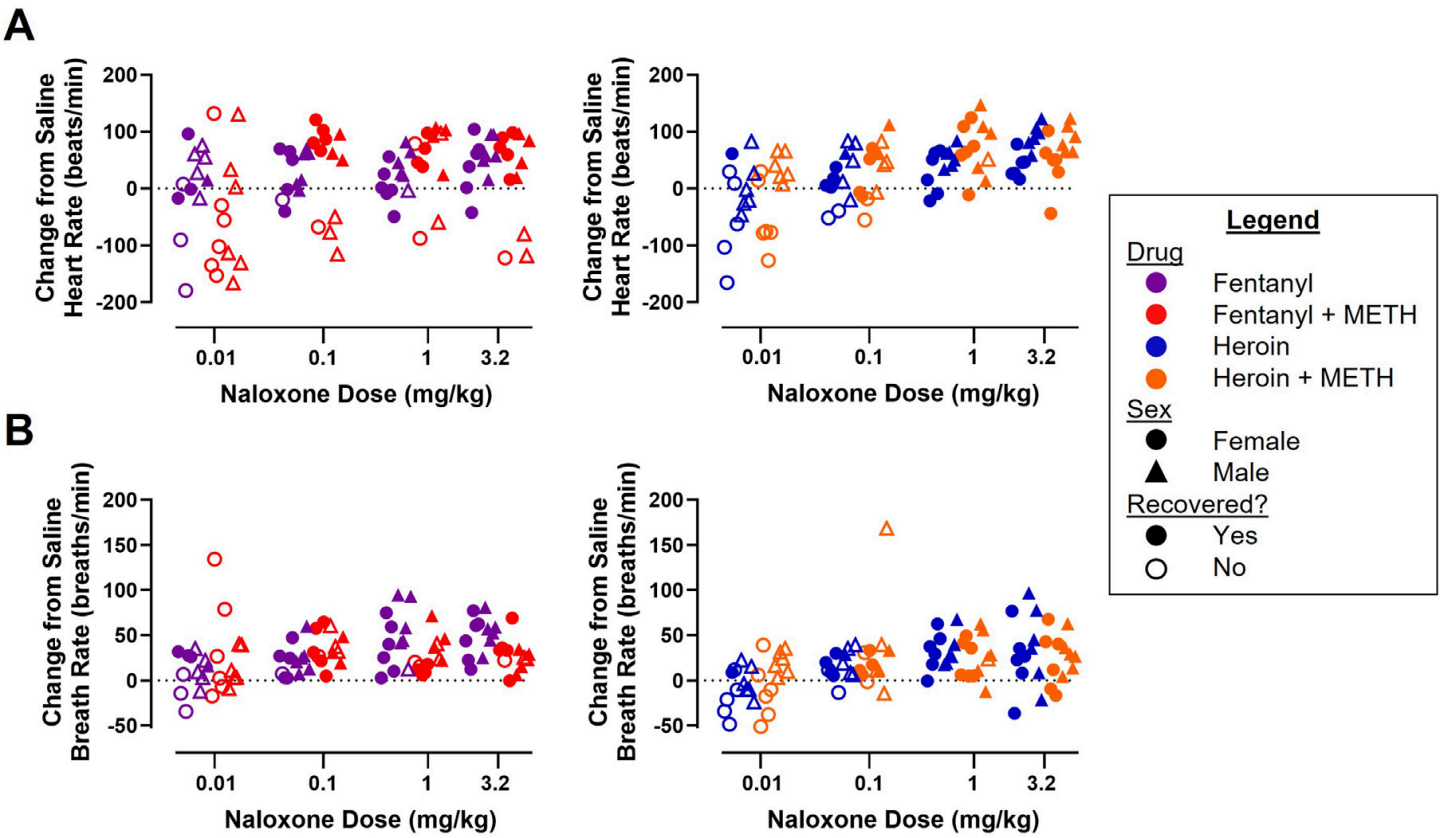
Effects of naloxone (0.01–3.2 mg/kg) administration on heart rate **(A)** and breath rate **(B)** following reversal of fentanyl (0.56 mg/kg) and 0.56 mg/kg fentanyl +1 mg/kg METH (left) and heroin (5.6 mg/kg) and 5.6 mg/kg heroin +1 mg/kg METH (right). Data represent the maximum HR or BR value recorded within 10 min after naloxone infusion minus the average HR or BR values over this same time frame (5–15 min) when the same subject was tested saline for both infusions (n = 6/sex).

A dose-effect function was established to evaluate the rebound tachycardia (Figure 5A) and tachypnea (Figure 5B) observed following naloxone (0.01–3.2 mg/kg) reversal of cardiorespiratory depression from fentanyl alone and fentanyl + METH (Figure 5, left) and from heroin alone and heroin + METH (Figure 5, right). Three-way ANOVAs for heroin alone and heroin + METH revealed a main effect of naloxone dose (F [3, 80] = 15.60; p < 0.0001) and sex (F [1, 80] = 25.69; p < 0.0001) on rebound tachycardia and of naloxone dose (F [3, 80] = 6.18; p < 0.001) and sex (F [1, 80] = 5.67; p < 0.05) on tachypnea, with male rats exhibiting greater rebound effects on HR and BR than female rats. There were no significant effects of drug condition or any interactions. Three-way ANOVAs for fentanyl alone and fentanyl + METH revealed a main effect of naloxone dose on rebound tachycardia (F [3, 80] = 3.97; p < 0.05) and tachypnea (F [3, 80] = 3.44; p < 0.05); there were no significant effects of sex or drug condition, but there was a significant naloxone dose × drug interaction observed for tachypnea (F [3, 80] = 4.17; p < 0.01).

## 4 Discussion

Overdose deaths involving opioids and stimulants have been increasing for the past 10+ years in the United States. Available evidence suggests that multi-drug overdoses require larger and/or more frequent administration of naloxone, which increases the risk for adverse cardiovascular complications (Merigian, 1993; Lameijer et al., 2014; van Lemmen et al., 2023; Dahan et al., 2024). In addition, current treatment strategies are ineffective at safely reversing overdoses involving opioids and stimulants, which may be contributing to annual increases in fatal multi-drug overdoses (Friedman and Shover, 2023). The current study sought to address this knowledge gap by using a collar-based pulse oximetry system that allowed for the continuous and simultaneous recording of cardiorespiratory endpoints (e.g., SpO_2_, HR, BR) in awake, freely moving rats to determine the potency and effectiveness of naloxone to reverse cardiorespiratory depression from fentanyl and heroin, administered alone and in combination with METH. There were three main findings: 1) naloxone was more potent at reversing fentanyl-than heroin-induced cardiorespiratory depression but fully effective for both opioids, 2) combining METH with heroin did not alter the potency or effectiveness of naloxone to recover cardiorespiratory function, and 3) naloxone was less effective and potent at reversing fentanyl + METH than fentanyl alone. Secondary analyses indicate co-administration of METH with either heroin or fentanyl did not affect the magnitude of the rebound tachycardia or tachypnea associated with naloxone reversal of cardiorespiratory endpoints. In addition, our study did not reveal any major sex differences in the depressant effects of heroin or fentanyl alone, the potency or effectiveness of naloxone to reverse cardiorespiratory depression, or the rebound tachycardia or tachypnea from reversal of the opioids or opioid + stimulant mixtures. Altogether, our data show that naloxone was less potent and effective at reversing the cardiorespiratory effects of a mixture of fentanyl + METH than fentanyl alone, suggesting that current treatment strategies may be ineffective in safely reversing fentanyl-involved multi-drug overdoses.

Although it is well-established that cardiorespiratory depression is the primary cause of fatal opioid overdoses, this study characterized the effects of heroin and fentanyl on SpO_2_, HR, and BR to identify equieffective doses at producing hypoxemia. Heroin and fentanyl dose-dependently increased the magnitude and duration of cardiorespiratory depression, with 0% of animals spontaneously recovering at the largest dose of heroin (5.6 mg/kg) and fentanyl (0.56 mg/kg). These data indicate that fentanyl is ∼10x more potent than heroin at producing hypoxemia. The doses of heroin and fentanyl assessed for naloxone reversal were chosen based on their ability to decrease SpO_2_ to a comparable magnitude for the entire 30-min experimental session. Our data further demonstrate that heroin- and fentanyl-induced cardiorespiratory depression were dose-dependently reversed following naloxone (0.01–3.2 mg/kg) administration. Furthermore, naloxone was fully effective at reversing cardiorespiratory depression from heroin and fentanyl but was ∼3-fold more potent for fentanyl than heroin. From our results, it was unexpected that naloxone was more potent for fentanyl than heroin given that clinical reports suggest that larger and more frequent doses of naloxone are often required to reverse overdoses involving fentanyl (van Lemmen et al., 2023; Dahan et al., 2024). However, the dose of the opioid administered cannot be controlled in emergency settings, and blood levels of fentanyl associated with overdose may be significantly larger than those achieved in the present study (Palamalai et al., 2013). Furthermore, overdose patients likely have additional psychoactive substances in their systems (e.g., METH, xylazine) that may not be reported, but can nonetheless complicate reversal or reduce the potency of naloxone in clinical settings (Pergolizzi Jr et al., 2021b; Friedman et al., 2022; Kiyatkin and Choi, 2024). In addition, the depressant effects of heroin on HR and BR were slightly more prolonged at the largest dose than fentanyl, suggesting that doses of 5.6 mg/kg heroin and 0.56 mg/kg fentanyl might not have been functionally equivalent in terms of their effects on overall cardiorespiratory function which could have also contributed to the slight differences in naloxone potency. Contrary to our results, a study using whole-body plethysmography in rats found that naloxone was equipotent and effective at reversing cardiorespiratory depression from doses of heroin (3.2 mg/kg) and fentanyl (0.1 mg/kg) (Hiranita et al., 2023). However, it should be noted that the dose of fentanyl was not only smaller than the dose used in our study, but also one of the smaller doses of fentanyl (0.05–0.3 mg/kg) that has previously been shown to produce muscle rigidity in anesthetized and mechanically ventilated rats (Lui et al., 1989; Fu et al., 1994; Haouzi and Tubbs, 2022). Furthermore, it has been suggested that these unique properties of fentanyl (e.g., chest wall rigidity, vocal cord closure) may reduce the potency or effectiveness of naloxone to reverse cardiorespiratory depression (Torralva and Janowsky, 2019; Miner et al., 2021) due to the involvement of noradrenergic mechanisms (Jerussi et al., 1987; Lui et al., 1989; Lui et al., 1993; Weinger et al., 1989; Weinger et al., 1995; Weinger and Bednarczyk, 1994). Taken together, our data do not support our hypothesis that naloxone is less potent and effective at reversing cardiorespiratory depression from fentanyl alone than heroin alone.

Clinical evidence suggests that larger and/or more frequent naloxone doses are required to reverse multi-drug overdoses involving opioids (Van Lemmen et al., 2023; Dahan et al., 2024). In the present study, naloxone was equipotent and fully effective at reversing cardiorespiratory depression from heroin alone and a mixture of heroin + METH. However, when naloxone was evaluated in rats treated with a mixture of fentanyl + METH, it was found to be both less potent and less effective at reversing cardiorespiratory depression as compared to rats treated with fentanyl alone. Whereas naloxone was effective at recovering SpO_2_ levels in 100% of rats treated with fentanyl, heroin, or heroin + METH, it was only able to recover baseline SpO_2_ in 75% of rats treated with a mixture of fentanyl + METH. These findings indicate that naloxone was less effective at reversing cardiorespiratory depression from fentanyl when METH was co-administered, which is consistent with studies suggesting a reduced effectiveness of naloxone in treating fentanyl-involved multi-drug overdoses (Pergolizzi Jr et al., 2021b; Cano et al., 2025). Contrary to our findings, Hiranita et al. (2023) found that METH did not affect the potency or effectiveness of naloxone to reverse cardiorespiratory depression from fentanyl or heroin. However, in addition to using smaller doses of opioid (0.1 mg/kg fentanyl and 3.2 mg/kg heroin) than were used in our study, Hiranita and colleagues only evaluated the effects of naloxone against mixtures of opioids + METH that included a very small dose of 0.1 mg/kg METH that was without effect when tested alone, and smaller than those that have been reported to increase cardiovascular function in rats (Arora et al., 2001; Hassan et al., 2016). Adverse cardiovascular effects of METH (e.g., hypertension, tachycardia) result from a hyperadrenergic state due to increased norepinephrine release from the locus coeruleus, similar to the noradrenergic effects of fentanyl (Schindler et al., 1992; Ferrucci et al., 2013; Torralva and Janowsky, 2019). In addition, naloxone-precipitated withdrawal can result in increased activity of noradrenergic neurons in the locus coeruleus, which mediates the rebound cardiovascular and respiratory effects (Chang and Dixon, 1990; Feria et al., 1990; Brent and Chahl, 1991; Maldonado, 1997). Similar to the doses of fentanyl and METH evaluated by Hiranita et al. (2023), the authors also administered smaller doses of naloxone (>0.1 mg/kg) than those employed in the current study, which may not have elicited as large of rebound effects as those observed in this study. Taken together, our findings support our hypothesis that naloxone is less potent and effective at reversing cardiorespiratory depression from fentanyl + METH than fentanyl alone.

While it was expected that naloxone would be less potent and effective at reversing cardiorespiratory depression from fentanyl when METH was co-administered, it was unexpected that rebound cardiorespiratory effects were not altered by METH involvement. Clinical data indicate that larger naloxone doses, commonly administered for fentanyl and multi-drug overdoses, increase the risk for cardiovascular complications (Merigian, 1993; Lameijer et al., 2014; Yugar et al., 2023). The overlapping effects of fentanyl, METH, and naloxone reversal of cardiorespiratory depression on noradrenergic signaling could contribute to a sympathomimetic toxidrome and explain the cardiovascular consequences (e.g., pulmonary edema, ventricular tachycardia, atrial fibrillation) observed following naloxone reversal of fentanyl-involved multi-drug overdoses (Lui et al., 1989; Chang and Dixon, 1990; Schindler et al., 1992; Torralva and Janowsky, 2019; Neumann et al., 2023; Kwok et al., 2024). Additionally, co-administration of naloxone with pharmacological treatments that reduce norepinephrine (e.g., α_2_-adrenergic receptor agonists) may alleviate adverse effects during multi-drug overdoses (e.g., METH-induced agitation) (Tackett et al., 2024). As found in our study, 100% of animals recovered from fentanyl, heroin, and heroin + METH but only 75% of animals recovered from fentanyl + METH. Although our findings support that larger naloxone doses produce greater rebound tachycardia and tachypnea following reversal of cardiorespiratory depression, METH co-administration did not further exacerbate HR or BR. Taken together, our data suggest that rebound tachycardia and tachypnea are not directly responsible for the reduced potency and effectiveness of naloxone to reverse cardiorespiratory depression from fentanyl when combined with METH.

Although a disproportionate number of men suffer from fatal opioid overdoses compared to women (Kaplovitch et al., 2015; Hoopsick et al., 2021; National Institute on Drug Abuse, 2024), this is not necessarily due to sex-related differences in the cardiorespiratory effects of opioids or their reversal by naloxone. Despite other preclinical and clinical studies reporting small sex-related differences in the cardiorespiratory effects of heroin, and sometimes fentanyl (Cruz and Rodríguez-Manzo, 2000; Overdyk et al., 2014; Brant et al., 2018; Herzig et al., 2020; Khanna et al., 2020; Garrett et al., 2021; Marchette et al., 2021; Marchette et al., 2023; Haile et al., 2022; Little and Kosten, 2023), we did not detect any major sex differences in the depressant effects of heroin or fentanyl on SpO_2_, HR, or BR. There is also mixed evidence to support sex-related differences in naloxone-precipitated and spontaneous withdrawal severity (Cicero et al., 2002; Little and Kosten, 2023; Carlson et al., 2024); however, we did not observe any major sex-related differences in the potency and effectiveness of naloxone to reverse the effects of opioids alone, or combined with METH. Our data do suggest that sex-related differences may exist in the potency of naloxone to reverse cardiorespiratory depression, the time to recovery, or the severity of rebound effects under some conditions (e.g., heroin, heroin + METH). However, it is unclear whether these slight differences would translate to meaningful differences in the potency or effectiveness of naloxone to reverse opioid overdoses in people or in the occurrence of adverse effects following naloxone reversal.

Although pulse oximetry provided reliable and continuous measures of SpO_2_, HR, and BR, reliance on these three endpoints is also a limitation of our study design as they do not capture the full range of cardiorespiratory effects of opioids, stimulants, or the reversal of cardiorespiratory depression by naloxone. However, pulse oximetry was chosen instead of other techniques such as plethysmography or blood gas analysis because it provided a relatively high throughput and non-invasive method for continuously monitoring multiple cardiorespiratory endpoints in awake, freely moving animals. Importantly, the MouseOx^®^ Plus system has been validated against other methods (e.g., co-oximeter, blood gas analyses) and provides accurate measures of SpO_2_ (Strohl et al., 2007; Gamble, 2016). However, further validation studies should be conducted to confirm the accuracy of the MouseOx^®^ Plus system at recording low SpO_2_ levels (<60%) in both rats and mice. Although reliance on measures of SpO_2_ limits mechanistic interpretations that could be made with other methods, it provides a clinically-relevant, functional readout of respiratory depression. In addition, our findings show clear tachypnea and tachycardia following naloxone reversal of opioid-induced cardiorespiratory depression, outcomes that are consistent with the literature of adverse effects that can occur during naloxone reversal (Feria et al., 1990; Kanof et al., 1992; Hunter, 2005; Lameijer et al., 2014). Because our data suggest that co-use of fentanyl and METH may introduce or exacerbate other cardiovascular or respiratory interactions during naloxone reversal of cardiorespiratory depression, future studies will utilize cardiovascular telemetry, electromyography, and/or laryngoscopy to determine the extent to which other physiological endpoints (e.g., hypertension, arrythmia, apnea, chest wall rigidity, vocal cord closure) contribute to the reduced potency and effectiveness of naloxone to reverse fentanyl-induced cardiorespiratory depression when METH is co-administered. Because some of these effects (e.g., chest wall rigidity and vocal cord closure) are thought to be mediated by non-mu-opioid receptors, future studies will evaluate adjunct treatments (e.g., α_2_-adrenergic receptor agonists, α_1_-adrenergic receptor antagonists) to probe mechanistic hypotheses and ultimately improve the effectiveness of naloxone to recover cardiorespiratory function following large doses of fentanyl and other commonly co-used drugs, such as METH. In addition, hypoxic brain injury following non-fatal opioid overdose can lead to long-term neurological consequences (e.g., amnesia, cognitive decline) (O’Brien and Todd, 2009; Voronkov et al., 2021; Winstanley et al., 2021; Winstanley et al., 2021; Christine et al., 2025; Mahoney et al., 2023). As such, future studies will utilize behavioral assays such as rotarod or Barnes maze to assess neurological damage following naloxone reversal of multi-drug overdoses.

In summary, the present data support our hypothesis that naloxone is less potent and effective at reversing cardiorespiratory depression from fentanyl + METH but failed to provide evidence to suggest that METH exacerbates the rebound tachycardia or tachypnea observed following naloxone reversal of fentanyl or heroin. These data suggest that unique properties of fentanyl (e.g., chest wall rigidity) may interact with other rebound effects from naloxone reversal of multi-drug overdoses involving opioids and stimulants to reduce the potency and effectiveness of naloxone to recover normal cardiorespiratory function. Altogether, these findings support the need for the identification and development of treatment strategies to safely and effectively reverse multi-drug overdoses involving fentanyl and stimulants, such as METH.

## Data Availability

The raw data supporting the conclusions of this article will be made available by the authors, without undue reservation.
